# Effects of High Ammonium Loading on Two Submersed Macrophytes of Different Growth Form Based on an 18-Month Pond Experiment

**DOI:** 10.3389/fpls.2022.939589

**Published:** 2022-07-14

**Authors:** Qing Yu, Haijun Wang, Hongzhu Wang, Chao Xu, Miao Liu, Yu Ma, Yan Li, Shuonan Ma, David P. Hamilton, Erik Jeppesen

**Affiliations:** ^1^State Key Laboratory of Freshwater Ecology and Biotechnology, Institute of Hydrobiology, Chinese Academy of Sciences, Wuhan, China; ^2^School of Life Sciences, Institute of Ecology and Biodiversity, Shandong University, Qingdao, China; ^3^School of Ecology and Environmental Science, Institute for Ecological Research and Pollution Control of Plateau Lakes, Yunnan University, Kunming, China; ^4^College of Fisheries and Life Science, Dalian Ocean University, Dalian, China; ^5^School of Marine Sciences, Ningbo University, Ningbo, China; ^6^Australian Rivers Institute, Griffith University, Nathan, QLD, Australia; ^7^Department of Ecoscience, WATEC, Aarhus University, Aarhus, Denmark; ^8^Sino-Danish Center for Education and Research, Beijing, China; ^9^Limnology Laboratory, Department of Biological Sciences, Middle East Technical University, Ankara, Turkey; ^10^Centre for Ecosystem Research and Implementation, Middle East Technical University, Ankara, Turkey; ^11^Institute of Marine Sciences, Middle East Technical University, Erdemli, Turkey

**Keywords:** pond experiment, growth form, submersed macrophytes, ammonium, population development

## Abstract

Ammonium (NH_4_-N) produces a paradoxical effect on submersed macrophytes because it is not only the preferred nitrogen source for the growth of plants but also threatens the growth of plants at high concentration. Whether short-term and small-scale physiological toxicity experiments at an individual level can reflect the effects of high ammonium on populations of submersed macrophytes in natural conditions is still unclear. In this study, an 18-month experiment was conducted in six 600 m^2^ ponds subjected to different levels of ammonium loading. The effects of high ammonium on populations of canopy-forming *Myriophyllum spicatum* and rosette-forming *Vallisneria natans* were explored. The results showed that *M. spicatum* and *V. natans* populations can develop high cover and height at high ammonium concentration (7 mg/L) at short-term exposures, and *V. natans* may be tolerant to 18 mg/L ammonium concentration. However, the cover of *M. spicatum* and the height of both species were inhibited at 2.4 mg/L at long-term exposures. The height of *M. spicatum* was two to six times higher than that of *V. natans* across all treatments and control by the end of the experiment, and the cover of *M. spicatum* was 7–11 times higher than that of *V. natans* in most NH_4_-N loading treatments, except the cover of *M. spicatum* in the highest NH_4_-N loading treatment with 18 mg/L NH_4_-N. The rosette-forming *V. natans* resists ammonium stress by slow growth (shoot elongation) to reduce consumption, while canopy-forming species resist ammonium stress by shoot elongation and canopy development to capture light. Although increasing ammonium concentration may induce severe stress on *M. spicatum*, the morphological characteristics of this species may, to some extent, release the plants from this stress. Our present study indicates that the negative effects of ammonium stress on the development of populations increased with exposure duration, and the submersed macrophyte community with stronger ability for light capture and dispersal may resist high ammonium stress. Nevertheless, in strongly ammonium-enriched systems, competition and succession cannot be neglected.

## Introduction

Due to their complex external morphology, submersed macrophytes create habitat heterogeneity in aquatic ecosystems, and therefore play an important role in the structure and function of shallow lakes (Jeppesen et al., 1998; Su et al., 2019). However, submersed macrophytes are experiencing a global decline in shallow lakes, resulting in eventually a shift from a macrophyte-dominated healthy ecosystem state to a phytoplankton-dominated state (Scheffer et al., 1993; Chao et al., 2022) and the deterioration of various functions of aquatic ecosystems (Sayer et al., 2010; Zhang et al., 2017). Therefore, to explore the responses of submersed macrophytes to the stressors is necessary for the macrophyte restoration in the management of lake ecosystems.

An array of stressors underlying the loss of submersed macrophytes have been identified, such as herbivore grazing (Mormul et al., 2018), extreme precipitation (Cobbaert et al., 2014), shading by phytoplankton in the process of eutrophication due to high loading of nutrients such as ammonia nitrogen and phosphorus (Søndergaard et al., 2017), their interactive effects under a background of climate change (Moss et al., 2011; Short et al., 2016), and heavy metal, such as cadmium and copper (Krayem et al., 2016; Bai et al., 2018). Among them, elevated levels of ammonium receive increasing attention (Shi et al., 2020; Xian et al., 2020), because the excessive input of ammonia nitrogen to aquatic ecosystems is caused by nitrogen deposition (Ti et al., 2018). Ammonium (NH_4_-N, including the ionic form NH_4_^+^ and aqueous NH_3_) is a paradoxical nutrient because it is not only the preferred nitrogen source for the growth of submersed macrophytes (Bornette and Puijalon, 2011) but also damages submersed macrophytes at a high concentration. The impacts associated with ammonium toxicity include (1) physiological changes, such as reactive oxygen species (ROS) accumulation (Nimptsch and Pflugmacher, 2007), imbalance of nitrogen/carbon metabolism (Zhang et al., 2011), and decline in photosynthesis (Shi et al., 2020); and (2) morphological changes, mainly decreased shoot height (or leaf length) (Saunkaew et al., 2011), biomass, and relative growth rate (Zhu Z. J. et al., 2018; Tan et al., 2019). It is evident from the abovementioned data that conclusions about the role of NH_4_-N in the decline of macrophytes in aquatic ecosystems have been almost exclusively based on physiological and morphological responses on individual plants, with exposures lasting from a few days up to 2 months, and conducted in aquaria of volume <1 m^3^. Great progress has been made in the previous studies of the mechanisms of ammonium on submersed macrophyte individuals. However, the stress from high NH_4_-N was most likely lower at higher shoot density because of direct uptake of NH_4_-N by the leaves and shoots (van der Heide et al., 2008), and a previous pond experiment found that high NH_4_-N had little or no effect on plant number (Yu et al., 2015). Therefore, based on the previous results, we argued that extrapolating short-term and small-scale experiments to large natural ecosystems may not give reliable or accurate results. It is necessary to explore the effects and mechanisms of high ammonium on the development of submersed macrophytes at a community level in natural conditions.

The effects and mechanisms of ammonium are closely related to the spatial and temporal scale of the experiments. For example, the pot experiments conducted in the ponds near natural conditions showed that high ammonium in winter and spring had a negative impact on the leaf length and leaf dry weight of *V. natans*, while it has a weak or even no impact on the number of plants and root dry weight; moreover, the tolerance of submersed macrophytes in summer and autumn is higher than that in winter and spring (Yu et al., 2015, 2017). The 11-month mesocosm experiment showed that long-term high nitrogen exposure had a negative impact on the growth of submersed macrophytes (Olsen et al., 2015). Thus, the effect of ammonium is related to exposure duration, and the sensitivity of submersed macrophytes to ammonium is also changeable in different growth periods (Ochieng et al., 2021). These results indicated that the previous small-scale toxicity experiments had focused on the responses of individual plants rather than the effects on the entire plant community and little was known about how high ammonium concentrations affect the development of submersed macrophytes at the community level. The previous experiments may have limited relevance to community succession in natural shallow lakes and have not extended over the lifetime of macrophyte development. Therefore, larger temporal-spatial scale studies are needed to explore the effects and mechanisms of high ammonium on the development of submersed macrophytes.

Growth form may also influence the responses of submersed macrophytes to the stress of high NH_4_-N loading. For example, canopy-forming and rosette-forming species are the two most common submersed macrophytes. Canopy-forming species form canopies on the water surface, whereas rosette-forming species spread under water through the formation of daughter ramet (He et al., 2019). The canopy-forming species have better access to light than rosette-forming species. Low light may aggravate NH_4_-related physiological stress, as suggested in previous short-term mesocosm experiments (Cao et al., 2011; Yuan et al., 2016; Zhu et al., 2018). This is because submersed macrophytes may synthesize free amino acids (FAAs) to avoid NH_4_-N toxicity when experiencing high NH_4_-N stress. On the one hand, this process is energetically expensive, and extra energy and carbohydrates are needed for the detoxication process (Bittsanszky et al., 2015), which may lead to the reduction of the macrophyte height and biomass growth. On the other hand, previous short-term experiments have found that high shoot density will alleviate the stress from high NH_4_-N (van der Heide et al., 2008), and plant number will not be inhibited by NH_4_-N stress (Yu et al., 2015). This suggests that rapid growth and strong alight capture ability at the community level may help submersed macrophyte populations to alleviate high NH_4_-N stress. Therefore, we hypothesized that the submersed macrophyte populations with stronger abilities for light capture and spread may resist high NH_4_-N stress.

To explore the responses of macrophytes to exposure to high NH_4_-N on a large temporal-spatial scale at the community level, two common and widely distributed submersed macrophytes were selected, namely, rosette-forming *Vallisneria natans* and canopy-forming *Myriophyllum spicatum*. In this study, the experiment was carried out in six experimental ponds (the average area was 600 m^2^, the water depth was 1.5 m), mimicking natural shallow lake ecosystems over 18 months, covering the entire life cycles of the two test macrophytes. To investigate the effects of NH_4_-N loading on the development of submersed macrophyte populations, six NH_4_-N loading treatments (N0: 0; N1: 5; N2: 11; N3: 22; N4: 36; N5: 50 kg/month, representing NH_4_-N concentrations of approximately 0, 2, 4, 8, 13, and 19 mg/L, respectively) were used without phosphorus addition. The objectives of the study were (1) to examine the effects of NH_4_-N on two coexisting populations of submersed macrophytes on the larger temporal-spatial scale and (2) to compare the responses of two growth forms of submersed macrophytes to the NH_4_-N additions at the community level.

## Materials and Methods

### Experimental Treatments

**FIGURE 1:**
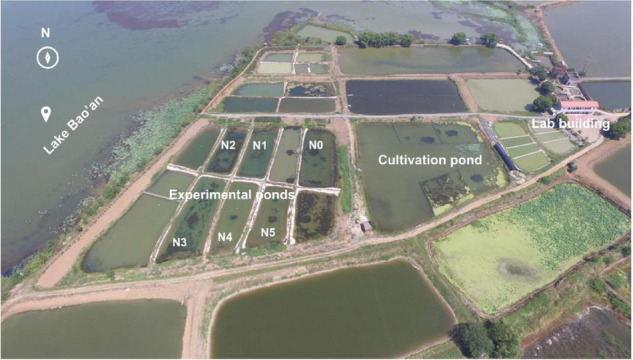
The experimental ponds and cultivation pond. NH_4_Cl fertilizer was added once per month from March 2017; the fertilization amount per month was 0 kg for N0, 5 kg for N1, 11 kg for N2, 22 kg for N3, 36 kg for N4, and 50 kg for N5. The photo was taken in June 2017.

The experimental ponds were located in the north-eastern part of Lake Bao’an (N37°17′17′′, E114°43′45′′) (Figure 1). The ponds had an average area of 600 m^2^ with a 1.5 m mean water depth and were constructed after dredging sediments. Water and sediments from Lake Bao’an were added to mimic the natural lake ecosystem.

On 15 September 2016, plants were collected from mesotrophic Lake Xi’liang (N29°58′30′′, E114°6′0′′, 72.1 km^2^ in area, 1.9 m in mean depth). Plants of similar size were chosen and cut to a leaf length of 15 cm before their introduction to a neighboring cultivation pond (Figure 1). On 25 December 2016, after 3 months of cultivation, the plants were cut again to a leaf length of 15 cm and introduced to the six experimental ponds (Figure 1), in an area of 2 m × 2 m, with bamboo poles marking the boundaries and water depth maintained at 30 cm to ensure sufficient light for macrophyte growth. The distance between individual plants was 10 cm, and the distance between *M. spicatum* areas and *V. natans* areas was 5 m. On 10 March 2017, the cover and height of submersed macrophytes were measured. Water levels were then increased to 1.6 m. Fertilization started on 20 March 2017, with additions of NH_4_Cl of 0 (N0), 5 (N1), 11 (N2), 22 (N3), 36 (N4), and 50 (N5) kg per month, which may ideally maintain a gradient of NH_4_-N concentration (i.e., 0, 2, 4, 8, 13, and 19 mg/L). The NH_4_Cl fertilizer (≥98.5% purity; Sinopharm Chemical Reagent Co., Ltd., Shanghai, China) was the only artificial nitrogen source and was dissolved in a polyethylene bucket with pond water for its application randomly across the surface of each fertilized pond. The experiment lasted for 18 months, from December 2016 to May 2018.

### Sampling and Measurements

The height of *M. spicatum* and *V. natans* was measured each month at 10 randomly selected sites in each pond. The areal cover (S_1_) of *M. spicatum* and *V. natans* and the water surface area of the pond (S_2_) were measured simultaneously to calculate the percentage plant cover:


$$\mathrm{Percentage}\,\mathrm{cover}=\frac{S_{1}}{S_{2}}\times 100\%$$

Environmental parameters were also measured every month. Water-dissolved oxygen (DO), pH, and conductivity (Cond) were measured *in situ* at 0.1 and 1.0 m depth with a YSI Pro Plus (Yellow Spring Inc., OH, Yellow Springs, United States). Water samples for chemical analysis were collected at five randomly chosen locations from each pond by integrating the water column with a tube sampler (1.5 m in height, 10 cm in diameter). Total nitrogen (TN) and total phosphorus (TP) were determined using spectrophotometry after digestion with K_2_S_2_O_8_ solution (Huang et al., 1999). Chlorophyll *a* of phytoplankton (Chl *a*) was determined by filtering a known volume of pond water through GF/C filters and extraction in 90% acetone at 4°C for 24 h (Whatman, GE Healthcare UK Limited, Buckinghamshire, United Kingdom). Absorbance values of the acetone extract were then read at two wavelengths (665 and 750 nm) by a spectrophotometer before and after acidification using 10% HCl, and calculations were used to obtain the Chl *a* concentration (Huang et al., 1999).

For analysis of ammonium (NH_4_-N), water was filtered using an acetate fiber microporous membrane (0.45 μm pore diameter; Shanghai Xingya Purification Material Factory, Shanghai, China), after which NH_4_-N was measured using Nessler’s reagent colorimetric method. The measured NH_4_-N concentration is total ammonia nitrogen (TAN, including both NH_4_^+^ and NH_3_). To estimate the relative concentrations of NH_4_^+^ and NH_3_ from TAN, the following equations were used (Emerson et al., 1975; Wood, 1993):


$$N\,H_{3}=N\,H_{4}-\frac{N\,H_{4}}{\left[1+10^{\left(p\,H-p\,K\,a\right)}\right]}$$

and


p⁢K⁢a=0.09018+2729.92/(273.2+Temp)

where, NH_4_-N, the concentration of ammonium (NH_4_-N, including both NH_4_^+^ and NH_3_) in mg/L; NH_3_, the concentration of un-ionized ammonia (NH_3_-N) in mg/L; pH, pH of the pond water; and Temp, temperature of the pond water in °C.

At the end of the experiment, about 0.5–1 g of fresh leaves from both species were ground in a 10% acetic acid solution to determine FAA. The filtered liquid was used to examine the FAA *via* the ninhydrin colorimetric method using leucine standards (Li et al., 2004).

### Statistical Analysis

The variables that did not follow normal distributions (Shapiro–Wilk test, *p* < 0.05) were log_10_ (*x*)-transformed, including TN, NH_4_-N, NH_3_, and height of populations. Pearson’s correlation was used to analyze the relationships between environmental variables and the height and cover of submersed macrophytes. SPSS 25, Microsoft Excel 2019, and R 4.1.2 (Rcore Team, 2020) were used to analyze the data. We conducted Seasonal and Regional Kendall trend tests to analyze long-term trends among different treatments (Jassby and Cloern, 2016). A Sen’s slope was considered significant when the associated *p*-value was <0.05. We also carried out Pettitt’s test to detect abrupt changes in submersed macrophyte populations (Pohlert, 2020).

## Results

### Nutrient Conditions in the Experimental Ponds

Throughout the pre-fertilization period (December 2016 to March 2017), no statistical differences among treatments were observed for TN, NH_4_-N, NH_3_, TP, Chl *a*, and pH (Supplementary Table 1). For the pre-fertilization period (3 months), the mean concentration of TN, NH_4_-N, and NH_3_-N in the six experimental ponds was <0.5, 0.2, and 0.01 mg/L, respectively. The corresponding mean concentrations of TP and Chl *a* were <0.01 mg/L and 1 μg/L, respectively.

**FIGURE 2:**
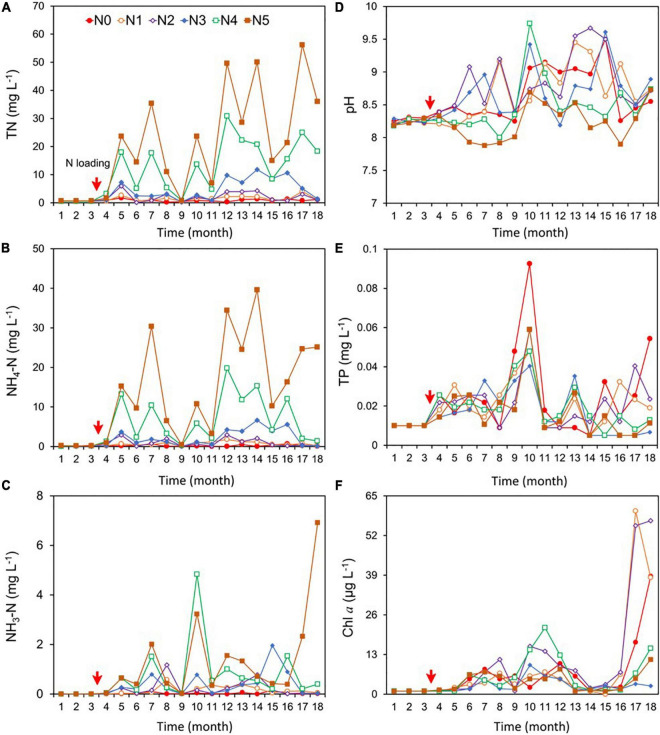
Changes in the concentrations of total nitrogen (TN) **(A)**, ammonium (NH_4_-N) (including both NH_3_ and NH_4_^+^) **(B)**, un-ionized ammonia (NH_3_-N) **(C)**, pH **(D)**, total phosphorus (TP) **(E)**, and phytoplankton chlorophyll *a* (Chl *a*) **(F)** during the experiment (December 2016–May 2018). NH_4_Cl fertilizer was added once per month from March 2017; the fertilization amount per month was 0 kg for N0, 5 kg for N1, 11 kg for N2, 22 kg for N3, 36 kg for N4, and 50 kg for N5. The red arrows indicate the start of fertilization, after which nitrogen fertilizer is applied monthly.

For the post-fertilization (N loading) period, the concentrations of the three nitrogen variables (TN, NH_4_-N, and NH_3_-N) varied with N treatment. TN and NH_4_-N were significantly higher in N1, N2, N3, N4, and N5 than in N0 (Figures 2A–C and Supplementary Table 1), with TN ranging between 0.8 and 27.0 mg/L, NH_4_-N between 0.2 and 18.0 mg/L, and NH_3_-N ranging between 0.01 and 6.9 mg/L. TN in N1, N4, and N5 showed a significant increasing trend over the 18-month study period (*p* < 0.05, Sen’s slope >0), and NH_4_-N showed a significant increasing trend in N1 and N5 (Supplementary Table 2). There was a clear increasing trend of NH_3_-N in N5 (Supplementary Table 2). The target gradient of NH_4_-N concentration based on loads was 0 (N0), 2 (N1), 4 (N2), 8 (N3), 13 (N4), and 19 (N5) mg/L; however, the actual concentration was 0.2, 0.8, 1.0, 2.4, 7.0, and 18.0 mg/L in the six respective treatments.

pH showed no significant difference in the N0 control and the N1, N2, N3, and N4 treatments, while pH was significantly lower in N5 than in N0–N4, with the mean for the 18 months ranging between 7.9 and 9.7 in all treatments (Figure 2D and Supplementary Table 1). The mean concentration of TP was 0.02 mg/L in each treatment (Figure 2E), with no significant trends among treatments during the 18 months (Supplementary Table 2).

Chlorophyll *a* showed no significant difference between N1, N2, N4, N5, and N0, but N0 was significantly higher than N3. Chl *a* concentration ranged between 4.0 and 14.0 μg/L in the six treatments (Figure 2F). Significant increasing trends of Chl *a* occurred in N0, N3, and N4 during the 18 months (Supplementary Table 2).

### Submersed Macrophyte Cover

**FIGURE 3:**
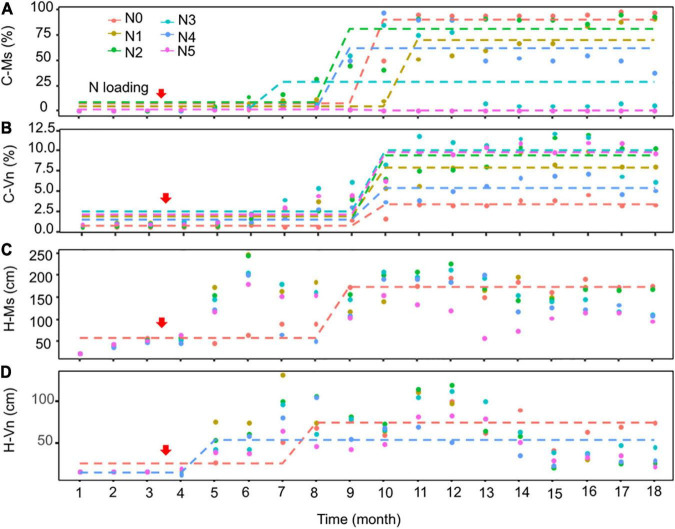
Pettitt’s test for single change-point detection for the cover (C-Ms, C-Vn, %) **(A,B)** and height (H-Ms, H-Vn, cm) **(C,D)** of *Myriophyllum spicatum* (Ms) and *Vallisneria natans* (Vn) for various treatments during the experiment. The dotted lines indicate that probable change point was detected (*p* < 0.05). The red arrows indicate the start of fertilization, after which nitrogen fertilizer is applied monthly. Refer to Figure 2 for the fertilization amounts of various treatment.

The Seasonal and Regional Kendall trend test indicated that the cover of *M. spicatum* and *V. natans* increased significantly (*p* < 0.05, Sen’s slope >0) during the 18 months, except for a decreasing trend of *M. spicatum* in N5 (*p* < 0.05, Sen’s slope <0) (Figures 3A,B and Supplementary Table 2). Pettitt’s test can detect single change-point in cover of both species in time series in all treatments. Most of the change-points occurred after 4–6 months of NH_4_-N loading.

**FIGURE 4:**
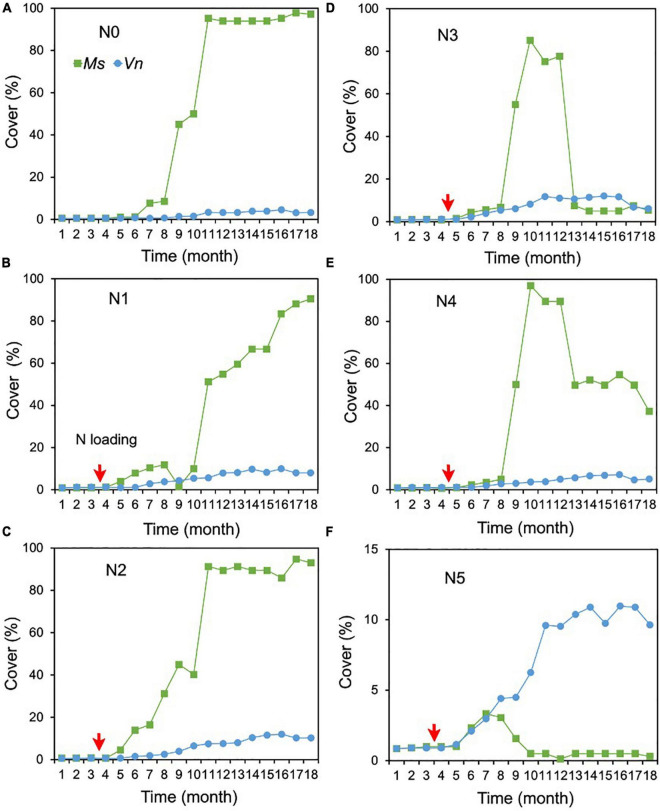
Changes in the cover of *Myriophyllum spicatum* (Ms) and *Vallisneria natans* (Vn) **(A–F)** for various treatments during the experiment. The red arrows indicate the start of fertilization, after which nitrogen fertilizer is applied monthly. Refer to Figure 2 for the fertilization amounts of various treatments.

For the cover of *M. spicatum*, there were no significant differences between N2 (1.0 mg/L), N3 (2.4 mg/L), N4 (7 mg/L), and N0, and the cover of *M. spicatum* was significantly higher in N0 than in N5 (with concentrations of 27 mg/L TN and 18 mg/L NH_4_-N) (Supplementary Table 1 and Figures 4A–F). In the N0–N2 treatments, the *M. spicatum* cover started to increase after 6 months of N loading and was >90% at the end of the experiment (Figures 4A–C). In the N3 (2.4 mg/L) and N4 (7 mg/L) treatments, the cover of *M. spicatum* was ∼90% during autumn 2017 after which a major drop occurred (Figures 4D,E).

As for the cover of *V. natans*, significant increasing trends were observed in all treatments (*p* < 0.05, Sen’s slope >0) during the 18 months (Figure 3B and Supplementary Table 2). The cover of *V. natans* in N1–N5 was significantly higher than in N0 (Supplementary Table 1). At the end of the experiment, the lowest cover (3.3%) was recorded in the N0 treatment (Figure 4A), and in the N1–N5 treatments, cover ranged between 5 and 10% (Figures 4B–F).

At the end of the experiment, the cover of *M. spicatum* in N1–N4 was 7–11 times higher than that of *V. natans*, while the cover of *M. spicatum* in N5 was lower than that of *V. natans* (Figure 4). The results of Pettitt’s test showed that the change points of *M. spicatum* cover were earlier than those of *V. natans* in N2–N5, while the change point was downward in N5 and upward in N2–N4 (Figures 3A,B).

### Submersed Macrophyte Height

**FIGURE 5:**
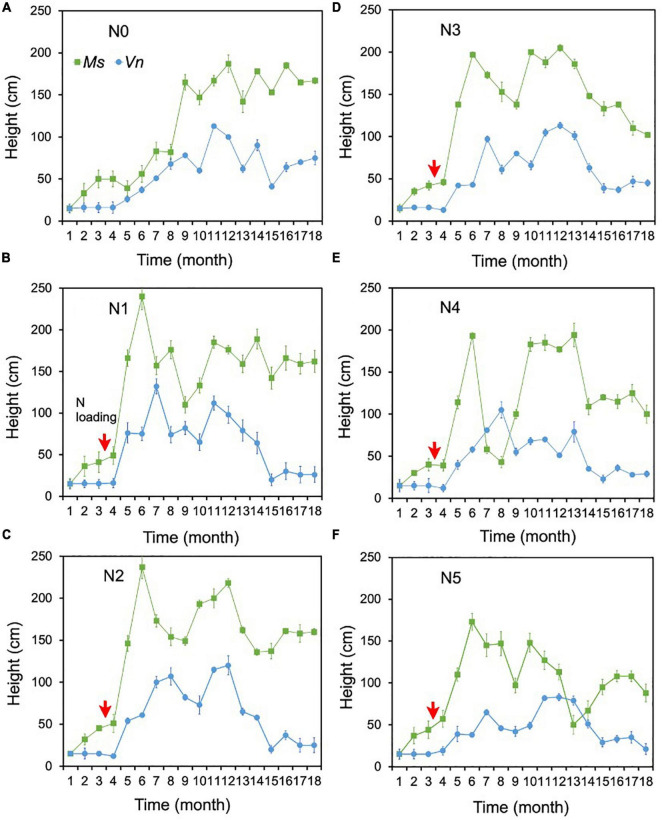
Changes in the heights of *Myriophyllum spicatum* (Ms) and *Vallisneria natans* (Vn) **(A–F)** for treatments during the experiment. The red arrows indicate the start of fertilization, after which nitrogen fertilizer is applied monthly. Refer to Figure 2 for the fertilization amounts of various treatment.

There were no change points identified for the height of the two species in most treatments using Pettitt’s test, except for the height of *M. spicatum* in N0 and the height of *V. natans* in N0 and N4 (Figures 3C,D). The height of *M. spicatum* and *V. natans* mostly leveled off or decreased in all treatments during the 18 months (Figures 5A–F).

The height of *M. spicatum* showed no significant difference between N1–N5 (i.e., for concentrations up to 18 mg/L NH_4_-N) and N0, although they increased across all treatments over the duration of the experiment. The height in N1–N3 was significantly greater than in N4 and N5 (Supplementary Table 1). The height of *M. spicatum* showed a clear increasing trend (*p* < 0.05, Sen’s slope >0) from the initial value of 15–167 cm in N0 (Figure 5A and Supplementary Table 2). In N1–N5, despite no significant trends (*p* > 0.05), the height still increased to greater than the initial value of 15 cm (Supplementary Table 2 and Figures 5B–F). However, the height of *M. spicatum* showed declining trends in N3–N5 treatments in the last few months of the experiment.

For *V. natans*, there were no significant differences in height in N1–N4 (with concentrations <7 mg/L NH_4_-N) and N0, while the height in N5 was significantly greater than in N0 (Supplementary Table 1 and Figures 5A–F). A clear increasing trend (*p* < 0.05, Sen’s slope >0) of *V. natans* occurred in N0 compared with the initial values of 15 cm, while the height in other treatments generally declined from 13 months (Supplementary Table 2 and Figures 5B–F).

The height of *M. spicatum* was two to six times higher than that of *V. natans* across all treatments and control by the end of the experiment.

### Relationships Between Submersed Macrophytes and Environmental Variables

**FIGURE 6:**
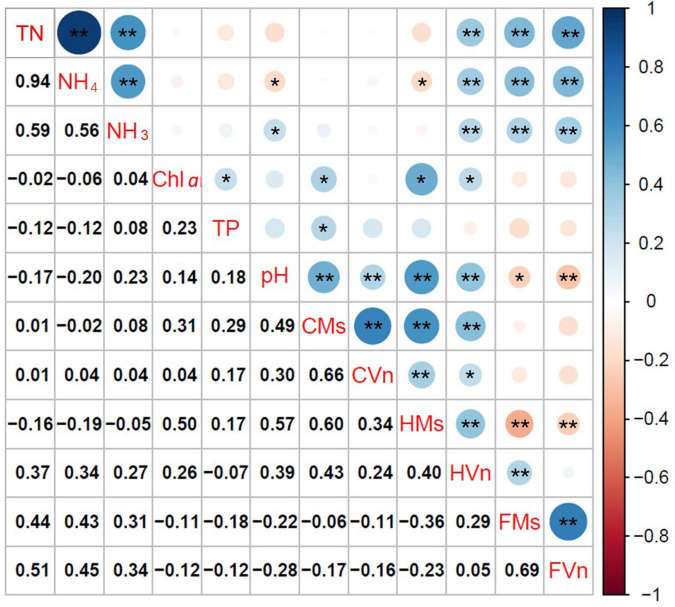
Coefficients of Pearson rank correlation of free amino acid content (FAA, mg/g), height (H, cm), and cover (C, %) of *Myriophyllum spicatum* (Ms) and *Vallisneria natans* (Vn), with the key environmental variables (*p < 0.05, **p < 0.01). TN, total nitrogen; NH_4_, ammonia nitrogen expressed as an ionized form; NH_3_, ammonia nitrogen expressed as an un-ionized form; Chl *a*, chlorophyll *a* of phytoplankton; TP, total phosphorus; CMs, cover of *M. spicatum*; CVn, cover of *V. natans*; HMs, height of *M. spicatum*; HVn, height of *V. natans*; FMs, FAA of *M. spicatum*; FVn, FAA of *V. natans*.

Figure 6 illustrates the relationships among TN, NH_4_-N, NH_3_-N, pH, Chl *a*, FAA, and the height and cover of *M. spicatum* and *V. natans* at the end of the experiment. The cover of *M. spicatum* was positively related (*p* < 0.05) to Chl *a*, TP, and pH, and the height of *M. spicatum* was negatively related to NH_4_-N, while positively related to pH and Chl *a* (Figure 6). The relationship between the cover of *V. natans* and pH was positively related, and the height of *V. natans* was positively related to TN, NH_4_-N, NH_3_-N, Chl *a*, and pH.

Free amino acids of both plants accumulated with increasing TN, NH_4_-N, and NH_3_-N (*p* < 0.05), but no significant relationships were established between cover and FAA. The relationships between FAA of *M. spicatum* and *V. natans* and pH were negatively correlated.

## Discussion

Clear seasonal dynamics were observed for TP and phytoplankton Chl *a* in the ponds, being higher in the summer and autumn, likely reflecting higher summer growth of phytoplankton combined with the stronger release of phosphorus from the sediment during the warmer season (Søndergaard et al., 2003; Xie, 2006).

Our pond experiment is, to our knowledge, the first attempt to reveal the influence of nitrogen loading at the community level for submersed macrophytes. In contrast to the experiments that have focused on the physiology and morphology of individual plants (Wang et al., 2008; Apudo et al., 2016; Zhu et al., 2018; Gao et al., 2019), our 18-month study dealing with populations revealed that *M. spicatum* and *V. natans* tolerated short-term ammonium stress when NH_4_-N was <7 mg/L, while the development under long-term ammonium stress for both species occurred when NH_4_-N exceeded 2.4 mg/L. A decline in submersed macrophyte height has previously been recorded at lower NH_4_-N <2–6 mg/L, which has been attributed to the direct toxic effects of NH_4_-N and associated with the combined stress of high pH and low light (Cao et al., 2011; Netten et al., 2013; van Diggelen et al., 2016; Zhu et al., 2018). In the present study, at NH_4_-N of 7 mg/L (N4), the cover and height of both *M. spicatum* and *V. natans* increased after 4 months of NH_4_-N loading, and the cover and height of *V. natans* also increased at NH_4_-N of 18 mg/L (N5). Relationships between pH and cover of *M. spicatum* and *V. natans* were positively significant, and there were positive relationships between cover and height of *M. spicatum* and Chl *a*, suggesting that pH and phytoplankton biomass did not impede the growth of submersed macrophytes. In a short-term experiment (up to 5 weeks), the results showed that plants were tolerant to NH_4_-N at high shoot densities (van der Heide et al., 2008). We found almost no loss of cover or height in most of the ponds (N1–N4) with <7 mg/L NH_4_-N after 9 months, and only the cover of *M. spicatum* was suppressed in the N5 treatment with a concentration of 18 mg/L NH_4_-N. However, during the last 6 months of the experiment, the tolerance concentration of both species was reduced to 2.4 mg/L. Therefore, our larger temporal-spatial pond experiment results suggest that exposure duration is of key importance in determining the effect of ammonium stress, which increased with exposure duration.

Our findings differ from previous findings revealing that NH_4_-N has a negative effect at a concentration of 2–6 mg/L, which causes physiological toxicity in plants with exposure duration <10 months (Netten et al., 2013; Rao et al., 2018; Zhu et al., 2018). In N1–N4 of our experiment, pH was close to 9, which should enhance the physiological toxicity of NH_3_ (Caicedo et al., 2000; Körner et al., 2001; Gao et al., 2015), but the cover of both submersed macrophyte species showed increasing trends, and there was a significant positive correlation between pH and height and cover of both growth forms of submersed macrophytes. Moreover, we did not find significant relationships between FAA and the population cover and height of both submersed macrophytes through the marked accumulation of FAA in *M. spicatum* and *V. natans* at the end of the experiment. Our results are consistent with some other field studies that moderate to high concentrations of NH_4_-N do not inhibit the growth of submersed macrophytes by physiological changes related to toxicity (Olsen et al., 2015; Zhao et al., 2016; Yu et al., 2017). Furthermore, we did not find that accumulation of FAA is a useful indicator of macrophyte growth and the physiological stress of plants in our field study as otherwise seen in pot experiments (Cao et al., 2009a; Yuan et al., 2015; Rao et al., 2018).

Due to the marked inhibition in the cover of *M. spicatum* in N5 treatment, the higher height of *M. spicatum* than *V. natan*s at the highest NH_4_-N concentration (18 mg/L) and higher height and cover of *M. spicatum* than that of *V. natans* in N1–N4 treatments tend to support our hypothesis that the submersed macrophyte community with stronger abilities of light capture and spread may resist high ammonium stress. Generally, the canopy-forming species *M. spicatum* might resist NH_4_-N stress by shoot elongation to enhance light capture, while *V. natans* may resist NH_4_-N stress by slow elongation growth of shoots to reduce resource consumption. During the experiment, the population cover and height of *M. spicatum* in N0–N4 increased to a much greater extent than that of *V. natans*. When exposed to 7 mg/L NH_4_-N, the cover of *M. spicatum* was around seven times higher than that of *V. natans*, and the height of *M. spicatum* was three times higher than that of *V. natans*, suggesting greater resilience of *M. spicatum* to high NH_4_-N. These results can be attributed to the physiological and morphological characteristics of the two species. First, to prevent NH_4_-N accumulation, the major physiological pathway (GS/GOGAT cycle) of NH_4_-N assimilation is activated, which synthesizes FAA from NH_4_-N (Xian et al., 2020). In many cases, this may result in disrupted metabolism and high carbon requirements for internal NH_4_-N detoxification (Netten et al., 2013; Gao et al., 2020). When the NH_4_-N supply is high, the GS/GOGAT cycle is modified and adapted to increase FAA, leading to increased energy requirements and high demand for carbohydrates. Thus, carbon depletion is a mechanism behind NH_4_-N inhibition of growth (Cao et al., 2007, 2011). However, *M. spicatum* has another pathway than *V. natans*, for ammonia detoxification, catalyzed by glutamate dehydrogenase (GDH) under high NH_4_-N conditions (Gao et al., 2020; Xian et al., 2020). Moreover, *M. spicatum* forms canopy vegetation with leaves on the water surface, where there is sufficient light for the production of carbohydrates to assist plants to overcome NH_4_-N stress (Fu et al., 2012; Yu et al., 2018; Angove et al., 2020). Finally, *M. spicatum* can reproduce asexually using vegetative fragments (produced naturally or broken mechanically) and disperse to other areas of our experimental ponds, e.g., *via* waves, invertebrates, and human disturbance (Li, 2014). However, the disturbance caused by wind wave may have led to a decline in height of *M. spicatum* in the last several months of the experiment, as the shoots of *M. spicatum* exposed to high N loading become fragile and easily breakable (Zhu et al., 2018), and canopy-forming species, like *M. spicatum*, are most vulnerable to wind disturbance. In contrast, *V. natans* roots in sediment disperse by plagiotropic stolon and spread horizontally above the ground (Xiao et al., 2006). Therefore, the cover of *V. natans* in N0–N4 treatments was much lower than that of *M. spicatum*. These results concurred with experiments demonstrating that the canopy-forming species (i.e., *Potamogeton crispus*) is tolerant to NH_4_-N concentration (<7 mg/L) (Cao et al., 2009b; Yu et al., 2018). Although the cover of *V. natans* keeps growing in all NH_4_-N addition treatments, the height began to decline after 13 months of N loading. This suggests that the rosette-forming species resist NH_4_-N stress by reducing the elongation growth of populations, i.e., reducing consumption to resist stress. The results of the community level coincide with the physiological imbalance of nitrogen/carbon metabolism (Zhang et al., 2011; Yuan et al., 2015; Rao et al., 2018).

## Conclusion

**FIGURE 7:**
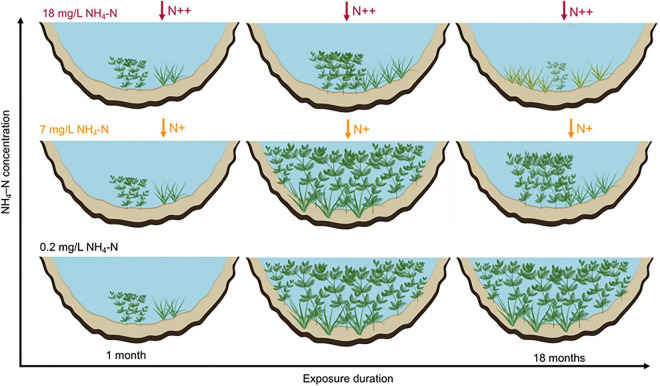
Schematic graphs showing the population development of canopy-forming *Myriophyllum spicatum* and rosette-forming *Vallisneria natans* in response to NH_4_-N concentrations at larger temporal-spatial scale.

We proposed a conceptual framework to describe the population development of submersed macrophytes responses to ammonium loading (Figure 7). The main conclusions are as follows: (1) populations of both growth forms can disperse in short-term exposure duration at 7 mg/L NH_4_-N, while the cover of *M. spicatum* and height of both species were inhibited at 2.4 mg/L in long-term exposures; (2) canopy-forming species can resist NH_4_-N stress through fast shoot elongation and canopy growth to capture light; (3) rosette-forming species can resist 18 mg/L NH_4_-N stress through reducing consumption; and (4) the negative effects of ammonium stress on the development of populations increased with exposure duration. Our findings have implications for decision-making on submersed macrophyte restoration in water with high N content. Both canopy-forming and rosette-forming species may potentially be used in the restoration of shallow lakes. However, canopy-forming species, if covering the water surface, may compete with rosette-forming submersed macrophyte populations for light and block their development and tolerance to high NH_4_-N concentration. These results point to the need to integrate interspecific competition with physiological stress indicators, morphological traits, and population characteristics, to better understand the mechanisms underlying macrophytes decline in shallow lakes. Our results also point to the importance of exposure duration to NH_4_-N loading, with lower thresholds of response at longer exposure time. The interactions between seasonal dynamics of submersed macrophyte populations and NH_4_-N or other environmental variables need to be further studied.

## Data Availability Statement

The original contributions presented in this study are included in the article/Supplementary Material, further inquiries can be directed to the corresponding authors.

## Author Contributions

QY: validation, conceptualization, investigation, methodology, formal analysis, data curation, and writing – original draft and review and editing. HJW: conceptualization, methodology, writing – review, supervision, resources, project administration, and funding acquisition. HZW: conceptualization, methodology, supervision, and project administration. CX, YM, and SM: validation and investigation. ML: validation, investigation, and data curation. YL: validation and writing – review and editing. DH: methodology and writing – review and editing. EJ: conceptualization, methodology, and writing – review and editing. All authors contributed to the article and approved the submitted version.

## Conflict of Interest

The authors declare that the research was conducted in the absence of any commercial or financial relationships that could be construed as a potential conflict of interest.

## Publisher’s Note

All claims expressed in this article are solely those of the authors and do not necessarily represent those of their affiliated organizations, or those of the publisher, the editors and the reviewers. Any product that may be evaluated in this article, or claim that may be made by its manufacturer, is not guaranteed or endorsed by the publisher.
