# Supplementary material for: Effects of High Ammonium Loading on Two Submersed Macrophytes of Different Growth Form Based on an 18-Month Pond Experiment
Source: Front Plant Sci. 2022 Jul 14;13:939589. doi: 10.3389/fpls.2022.939589 (PMC9330597; doi:10.3389/fpls.2022.939589)
Supplement: Supplementary file 1 [file Table_1.DOCX]

Table S1 Differences among treatments over the entire experimental period based on Friedman test (a) and Wilcoxon Signed-Rank Test (b) when the Friedman test gave a significant *p* value (*p*<0.05). TN, total nitrogen (mg L^-1^); NH_4_, ammonium nitrogen (mg L^-1^); NH_3_, ammonia nitrogen (mg L^-1^); TP, total phosphorus (mg L^-1^); Chl *a*, phytoplankton chlorophyll *a* (μg L^-1^); H, height of plants in cm; C, cover of plants in %; Ms, *Myriophyllum spicatum*; Vn, *Vallisneria natans*. A significant difference (*p-*value < 0.05) is shown in bold.

| (a) Friedman Test | | | | | | | | | | |
| --- | --- | --- | --- | --- | --- | --- | --- | --- | --- | --- |
|  | TN | NH_4_ | NH_3_ | TP | Chl *a* | pH | H-Ms | H-Vn | C-Ms | C-Vn |
| *p* | **<0.001** | **<0.001** | **<0.001** | **<0.001** | **<0.001** | **<0.001** | **<0.001** | **<0.001** | **<0.001** | **<0.001** |
| (b) Wilcoxon Signed-Rank Test | | | | | | | | | | |
| Pre-fertilization | | | | | | Post-fertililzation | | | | |
|  |  |  | N1 N2 N3 N4 N5 | | | N1 | N2 | N3 | N4 | N5 |
| *p* | TN | N0 |  |  |  | **0.02** | **0.03** | **0.001** | **0.001** | **<0.001** |
|  |  | N1 |  |  |  |  | 0.23 | **0.002** | **0.001** | **<0.001** |
|  |  | N2 |  |  |  |  |  | **0.003** | **0.001** | **<0.001** |
|  |  | N3 |  |  |  |  |  |  | **0.001** | **<0.001** |
|  |  | N4 |  |  |  |  |  |  |  | **0.001** |
| *p* | NH_4_ | N0 |  |  |  | **0.02** | 0.06 | **0.01** | **<0.001** | **<0.001** |
|  |  | N1 |  |  |  |  | 0.72 | **0.04** | **<0.001** | **<0.001** |
|  |  | N2 |  |  |  |  |  | **0.03** | **<0.001** | **<0.001** |
|  |  | N3 |  |  |  |  |  |  | **<0.001** | **<0.001** |
|  |  | N4 |  |  |  |  |  |  | **<0.001** | **<0.001** |
| *p* | NH_3_ | N0 |  |  |  | **0.018** | **0.002** | **0.002** | **<0.001** | **<0.001** |
|  |  | N1 |  |  |  |  | 0.586 | 0.472 | **0.002** | **0.001** |
|  |  | N2 |  |  |  |  |  | 0.616 | **0.01** | **0.004** |
|  |  | N3 |  |  |  |  |  |  | **0.006** | **0.01** |
|  |  | N4 |  |  |  |  |  |  |  | 0.133 |
| *p* | pH | N0 |  |  |  | 0.98 | 0.18 | 0.65 | 0.07 | **0.001** |
|  |  | N1 |  |  |  |  | 0.22 | 0.95 | **0.04** | **0.004** |
|  |  | N2 |  |  |  |  |  | 0.45 | **0.03** | **0.001** |
|  |  | N3 |  |  |  |  |  |  | **0.05** | **0.001** |
|  |  | N4 |  |  |  |  |  |  |  | **0.002** |
| *p* | TP | N0 |  |  |  | 0.42 | 0.33 | 0.12 | 0.31 | 0.06 |
|  |  | N1 |  |  |  |  | 0.97 | 0.12 | 0.49 | 0.13 |
|  |  | N2 |  |  |  |  |  | 0.33 | 0.80 | 0.16 |
|  |  | N3 |  |  |  |  |  |  | 0.12 | 0.95 |
|  |  | N4 |  |  |  |  |  |  |  | 0.41 |
| *p* | Chl *a* | N0 |  |  |  | 0.75 | 0.07 | **0.04** | 0.51 | 0.09 |
|  |  | N1 |  |  |  |  | **0.04** | 0.13 | 0.78 | 0.28 |
|  |  | N2 |  |  |  |  |  | **0.004** | 0.20 | **0.01** |
|  |  | N3 |  |  |  |  |  |  | **0.03** | 0.25 |
|  |  | N4 |  |  |  |  |  |  |  | 0.10 |
| *p* | C-Ms | N0 |  |  |  | **0.04** | 0.53 | 0.07 | 0.08 | **0.004** |
|  |  | N1 |  |  |  |  | **0.001** | 0.12 | 0.36 | **0.001** |
|  |  | N2 |  |  |  |  |  | **0.01** | **0.03** | **0.001** |
|  |  | N3 |  |  |  |  |  |  | 0.07 | **<0.001** |
|  |  | N4 |  |  |  |  |  |  |  | **0.01** |
| *p* | C-Vn | N0 |  |  |  | **<0.001** | **<0.001** | **<0.001** | **<0.001** | **<0.001** |
|  |  | N1 |  |  |  |  | 0.267 | **0.02** | **0.001** | **0.002** |
|  |  | N2 |  |  |  |  |  | **0.04** | **0.01** | 0.06 |
|  |  | N3 |  |  |  |  |  |  | **0.001** | **0.04** |
|  |  | N4 |  |  |  |  |  |  |  | **0.002** |
| *p* | H-Ms | N0 |  |  |  | 0.46 | 0.17 | 0.44 | 0.36 | 0.25 |
|  |  | N1 |  |  |  |  | 0.85 | 0.38 | **0.01** | **0.002** |
|  |  | N2 |  |  |  |  |  | 0.06 | **0.001** | **0.001** |
|  |  | N3 |  |  |  |  |  |  | **0.004** | **0.001** |
|  |  | N4 |  |  |  |  |  |  |  | 0.15 |
| *p* | H-Vn | N0 |  |  |  | 0.96 | 0.89 | 0.84 | 0.15 | **0.03** |
|  |  | N1 |  |  |  |  | 0.67 | 0.94 | 0.10 | **0.01** |
|  |  | N2 |  |  |  |  |  | 0.92 | 0.06 | **0.03** |
|  |  | N3 |  |  |  |  |  |  | **0.02** | **0.001** |
|  |  | N4 |  |  |  |  |  |  |  | 0.49 |
