# Supplementary material for: Effects of High Ammonium Loading on Two Submersed Macrophytes of Different Growth Form Based on an 18-Month Pond Experiment
Source: Front Plant Sci. 2022 Jul 14;13:939589. doi: 10.3389/fpls.2022.939589 (PMC9330597; doi:10.3389/fpls.2022.939589)
Supplement: Supplementary file 2 [file Table_2.DOCX]

Table S2 Long-term trends for various treatments based on the Seasonal and Regional Kendall trend test. A significant trend (*p*-value < 0.05) is shown in bold. See Figure 2 for the fertilization amounts of various treatments.

|  |  | N0 | N1 | N2 | N3 | N4 | N5 |
| --- | --- | --- | --- | --- | --- | --- | --- |
| TN | *p* | 0.22 | **0.04** | 0.68 | 0.68 | **0.04** | **0.04** |
|  | Sen’s slope | 0.41 | 0.99 | 0.72 | 7.42 | 12.77 | 24.71 |
| NH_4_ | *p* | 0.68 | 0.22 | 0.68 | 0.68 | 0.68 | **0.04** |
|  | Sen’s slope | 0.08 | 0.28 | 0.2 | 3.85 | 7.41 | 15.43 |
| NH_3_ | *p* | 0.22 | **0.04** | 0.68 | 0.68 | 0.22 | **0.04** |
|  | Sen’s slope | 0.01 | 0.1 | 0.08 | 0.4 | 0.38 | 1.02 |
| pH | *p* | 0.68 | **0.04** | 0.37 | **0.04** | **0.04** | 1.00 |
|  | Sen’s slope | 0.44 | 0.67 | 0.76 | 0.49 | 0.27 | 0.05 |
| TP | *p* | 1.00 | 0.68 | 0.68 | 0.22 | 0.68 | 0.68 |
|  | Sen’s slope | 0.00 | 0.00 | 0.00 | -0.01 | -0.01 | -0.01 |
| Chl *a* | *p* | **0.04** | 0.37 | 0.37 | **0.04** | **0.04** | 0.37 |
|  | Sen’s slope | 3.15 | 3.37 | 0.35 | 0.81 | 1.05 | 0.35 |
| C-Ms | *p* | **0.04** | **0.04** | **0.04** | **0.04** | **0.04** | **0.04** |
|  | Sen’s slope | 45.5 | 73.92 | 88.71 | 4.11 | 48.78 | -0.50 |
| C-Vn | *p* | **0.04** | **0.04** | **0.04** | **0.04** | **0.04** | **0.04** |
|  | Sen’s slope | 3.04 | 7.19 | 9.66 | 10.13 | 5.21 | 9.63 |
| H-Ms | *p* | **0.04** | 0.68 | 0.22 | 0.68 | 0.22 | 0.68 |
|  | Sen’s slope | 126.5 | 109 | 98 | 91.5 | 77.5 | 32.5 |
| H-Vn | *p* | **0.04** | 0.68 | 0.68 | **0.04** | 0.68 | 0.68 |
|  | Sen’s slope | 45.5 | 9.5 | 15 | 23.5 | 14 | 14 |
